# Supplementary material for: Efficacy of fluopyram applied by chemigation on controlling eggplant root-knot nematodes (Meloidogyne spp.) and its effects on soil properties
Source: PLoS One. 2020 Jul 6;15(7):e0235423. doi: 10.1371/journal.pone.0235423 (PMC7337292; doi:10.1371/journal.pone.0235423)

PLOS ONE

Efficacy of fluopyram applied by chemigation on controlling eggplant root-knot nematodes (Meloidogyne spp.)

--Manuscript Draft--

**Manuscript Number:**	PONE-D-20-00394
**Article Type:**	Research Article
**Full Title:**	Efficacy of fluopyram applied by chemigation on controlling eggplant root-knot nematodes (Meloidogyne spp.)
**Short Title:**	Efficacy of fluopyram applied by chemigation on controlling eggplant root-knot nematodes
**Corresponding Author:**	Wen-Wei Tang, PhD<br>Guangxi Key Laboratory of Agro-Environment and Agric-Product Safety<br>Nanning, CHINA
**Keywords:**	eggplant; root-knot nematode; fluopyram; chemigation; activity of soil enzyme; functional diversity of soil microbial community
**Abstract:**	The root-knot nematode is one of the major challenges in eggplant production. Fluopyram, known to be an effective fungicide, is also used for controlling root-knot nematode. However, in China, little information is currently available regarding the efficacy of fluopyram via chemigation against root-knot nematode and the its effects on soil properties. For this, the objective of this work was to test mortality of root-knot nematode, functional diversity of soil microbial community, activity of soil enzyme after fluopyram applicated by chemigation. The results of two field experiments revealed that concentration of 60 g/ha fluopyram applied with 200 L/ha irrigation water at 2 L/h flow velocity were the most effective chemigation parameters for controlling eggplant root-knot nematode. The functional diversity of the soil microbial community was significantly affected by fluopyram. The activities of soil urease and β - glucosidase decreased during the initial stages but recovered at later stages. In brief, fluopyram is advantageous for the efficient control of root-knot nematode with no deleterious effects on soil properties as well as chemigation is positive for application in karst landscape in Guangxi.
**Order of Authors:**	Jinzhao Li
	Cancan Wang
	Saqib Hussain Bangash
	Haiou Lin
	Wen-Wei Tang, PhD
	Dongqiang Zeng
**Additional Information:**	
**Question**	**Response**
**Financial Disclosure**<br><br>Enter a financial disclosure statement that describes the sources of funding for the work included in this submission. Review the submission guidelines for detailed requirements. View published research articles from *PLOS ONE* for specific examples.<br><br>This statement is required for submission	Dongqiang Zeng received the award supported by the Key Research and Development Program of Guangxi (Guike AB16380118).<br>The funders had no role in study design, data collection and analysis, decision to publish, or preparation of the manuscript.

Powered by Editorial Manager® and ProduXion Manager® from Aries Systems Corporation

and **will appear in the published article** if the submission is accepted. Please make sure it is accurate.<br><br>**Unfunded studies**<br>Enter: *The author(s) received no specific funding for this work.*<br><br>**Funded studies**<br>Enter a statement with the following details:<br>• Initials of the authors who received each award<br>• Grant numbers awarded to each author<br>• The full name of each funder<br>• URL of each funder website<br>• Did the sponsors or funders play any role in the study design, data collection and analysis, decision to publish, or preparation of the manuscript?<br>• **NO** - Include this sentence at the end of your statement: *The funders had no role in study design, data collection and analysis, decision to publish, or preparation of the manuscript.*<br>• **YES** - Specify the role(s) played.<br><br>* typeset	
**Competing Interests**<br><br>Use the instructions below to enter a competing interest statement for this submission. On behalf of all authors, disclose any competing interests that could be perceived to bias this work—acknowledging all financial support and any other relevant financial or non-financial competing interests.<br><br>This statement **will appear in the published article** if the submission is accepted. Please make sure it is accurate. View published research articles from *PLOS ONE* for specific examples.	The authors have declared that no competing interests exist.

**NO authors have competing interests**<br><br>Enter: *The authors have declared that no competing interests exist.*<br><br>**Authors with competing interests**<br><br>Enter competing interest details beginning with this statement:<br><br>*I have read the journal's policy and the authors of this manuscript have the following competing interests: [insert competing interests here]*<br><br>* typeset	
**Ethics Statement**<br><br>Enter an ethics statement for this submission. This statement is required if the study involved:<br><br>• Human participants<br>• Human specimens or tissue<br>• Vertebrate animals or cephalopods<br>• Vertebrate embryos or tissues<br>• Field research<br><br>Write "N/A" if the submission does not require an ethics statement.<br><br>General guidance is provided below. Consult the submission guidelines for detailed instructions. **Make sure that all information entered here is included in the Methods section of the manuscript.**	N/A

**Format for specific study types**<br><br>**Human Subject Research (involving human participants and/or tissue)**<br>• Give the name of the institutional review board or ethics committee that approved the study<br>• Include the approval number and/or a statement indicating approval of this research<br>• Indicate the form of consent obtained (written/oral) or the reason that consent was not obtained (e.g. the data were analyzed anonymously)<br><br>**Animal Research (involving vertebrate animals, embryos or tissues)**<br>• Provide the name of the Institutional Animal Care and Use Committee (IACUC) or other relevant ethics board that reviewed the study protocol, and indicate whether they approved this research or granted a formal waiver of ethical approval<br>• Include an approval number if one was obtained<br>• If the study involved *non-human primates*, add *additional details* about animal welfare and steps taken to ameliorate suffering<br>• If anesthesia, euthanasia, or any kind of animal sacrifice is part of the study, include briefly which substances and/or methods were applied<br><br>**Field Research**<br><br>Include the following details if this study involves the collection of plant, animal, or other materials from a natural setting:<br>• Field permit number<br>• Name of the institution or relevant body that granted permission	
**Data Availability**<br><br>Authors are required to make all data underlying the findings described fully available, without restriction, and from the time of publication. PLOS allows rare exceptions to address legal and ethical concerns. See the PLOS Data Policy and FAQ for detailed information.	Yes - all data are fully available without restriction

A Data Availability Statement describing where the data can be found is required at submission. Your answers to this question constitute the Data Availability Statement and **will be published in the article**, if accepted.<br><br>**Important:** Stating 'data available on request from the author' is not sufficient. If your data are only available upon request, select 'No' for the first question and explain your exceptional situation in the text box.<br><br>Do the authors confirm that all data underlying the findings described in their manuscript are fully available without restriction?	
**Describe where the data may be found in full sentences. If you are copying our sample text, replace any instances of XXX with the appropriate details.**<br><br>• If the data are **held or will be held in a public repository**, include URLs, accession numbers or DOIs. If this information will only be available after acceptance, indicate this by ticking the box below. For example: *All XXX files are available from the XXX database (accession number(s) XXX, XXX.).*<br>• If the data are all contained **within the manuscript and/or Supporting Information files**, enter the following: *All relevant data are within the manuscript and its Supporting Information files.*<br>• If neither of these applies but you are able to provide **details of access elsewhere**, with or without limitations, please do so. For example:<br><br>*Data cannot be shared publicly because of [XXX]. Data are available from the XXX Institutional Data Access / Ethics Committee (contact via XXX) for researchers who meet the criteria for access to confidential data.*<br><br>*The data underlying the results presented in the study are available from (include the name of the third party*	All relevant data are within the manuscript.

and contact information or URL).<br>• This text is appropriate if the data are owned by a third party and authors do not have permission to share the data.<br><br>* typeset	
Additional data availability information:	Tick here if the URLs/accession numbers/DOIs will be available only after acceptance of the manuscript for publication so that we can ensure their inclusion before publication.

Powered by Editorial Manager® and ProduXion Manager® from Aries Systems Corporation

Manuscript Click here to download Manuscript Manuscript.doc

Efficacy of fluopyram applied by chemigation on controlling eggplant root-knot nematodes (*Meloidogyne spp*.) and its effects on soil properties

Jinzhao Li, Cancan Wang, Saqib Hussain Bangash, Haiou Lin, Dongqiang Zeng, Wenwei Tang*

Guangxi Key Laboratory of Agric-Environment and Agric-Product Safety, Agricultural College, Guangxi University, Nanning 530005, Guangxi, People's Republic of China

*Corresponding author: E-Mail: wenweitg@163.com.

Tel: +86-0771-3231584, Fax: +86-0771-3231584

// Abstract

The root-knot nematode (*Meloidogyne spp.*) is one of the major challenges in eggplant (*Solanum melongena* L.) production. Fluopyram, known to be an effective fungicide, is also used for controlling root-knot nematode. However, in China, little information is currently available regarding the efficacy of fluopyram via chemigation against root-knot nematode and the its effects on soil properties. For this, the objective of this work was to test mortality of root-knot nematode, functional diversity of soil microbial community, activity of soil enzyme after fluopyram applicated by chemigation. The results of two field experiments revealed that concentration of 60 g·ha^{-1} fluopyram applied with 200 L·ha^{-1} irrigation water at 2 L·h^{-1} flow velocity were the most effective chemigation parameters for controlling eggplant root-knot nematode. The functional diversity of the soil microbial community was significantly affected by fluopyram. The activities of soil urease and β - glucosidase decreased during the initial stages but recovered at later stages. In brief, fluopyram is advantageous for the efficient control of root-knot nematode with no deleterious effects on soil properties as well as chemigation is positive for application in karst landscape in Guangxi.

Introduction

The root-knot nematode (RKN, *Meloidogyne spp*.) is one of the best known and most harmful plant parasitic nematodes that causes serious damage to important agricultural crops, particularly eggplant (*Solanum melongena*) [1,2]. RKN penetrates growing root tips and forms multinucleate giant cells in damaged tissues, leading to gall formation, resulting in forked and defective eggplants [3], and subsequently disrupting physiological processes [4]. The damage caused by RKN is more frequent during hot climatic conditions and results in massive losses in net productivity [5].

Fluopyram (*N*-[2-[3-chloro-5-(trifluoromethyl)-2-pyridyl]ethyl]-α, α, α - trifluoro-ortho-toluamide) was initially developed as a fungicide by Bayer Crop Science in 2012 and was mainly used to control grey mould and powdery mildew in grapes but was also used against fungi in many other fruits and crops [6–8]. Recently, it has been confirmed that fluopyram contains a succinate dehydrogenase inhibitor (SDHI), which can be used as nematicide [9]. Fluopyram is registered in China as nematicide for use in tomato by soil drenching. Although fluopyram is widely used to control nematode growth, the RKN control efficacy via chemigation has thus far been rarely reported.

Guangxi is a part of southwest karst region in China which area is about 5.5 million km^2, accounting for 15.97% of national karst area [10]. Karst area is dreadful for agriculture development with thin soil layer, faint ground water impact and serious water leaking [11]. To overcome the demerit, irrigation systems have been used successfully for vegetable production over many years. The sown area (SA) of major farm crops in Guangxi was 6.15 million ha in 2016, and the water-saving irrigation area (WSIA) was 1.03 million ha. Within this WSIA, the sprinkling-drip irrigation area (SDIA) was 0.1 million ha, the ratio of WSIA / SA was 16.77%, which increased by 8.12% from last year, and the ratio of SDIA / IA was 9.85%, which increased by 39.91% from last year [12].

In this study, 'chemigation' consists of installing a chemical bucket to the original drip irrigation system and can be a conveniently applied method because drip irrigation is widely used in farms in Guangxi. Initially, such irrigation systems were used as a water-saving technique, whilst they are currently used for fertilizer and insecticide applications worldwide [13–14]. The benefits of chemigation are many, and this method has reduced insect pest problems more than traditional foliar applications or other methods. It is reasonable to assume that fluopyram applied by chemigation is efficacious for RKN control. Many studies have examined the risks of pesticides to soil organisms.

Fluopyram was first developed as a fungicide, and it was confirmed to change soil microbial communities [15]. Furthermore, changes in the soil environment caused by fungicides usually lead to reductions in the abundance and diversity of microorganisms [16]. During the cycling of nutrients, some hydrolytic enzymes are involved (β-glucosidase, urease, and phosphatase linked to C, N, and P, respectively). These enzymes are sensitive indicators of changes in the soil properties and show a strong relationship with the content and quality of soil organic mulches. It is reasonable to assume that fluopyram affects soil health and productivity.

Therefore, the aim of the study was to enhance fluopyram efficacy against eggplant RKN by chemigation and to investigate the effects of fluopyram on the development of eggplant roots, the functional diversity of the soil microbial community, the activity of soil enzymes and the terminal residues of fluopyram in eggplant fruit to verify its safety for both eggplants and soil ecosystems. The study will also aid in promoting fluopyram for control of eggplant RKN by application via chemigation.

Materials and Methods

Instruments and reagents

The chemigation system was based on the available drip irrigation system (Jiejiarun Agriculture Technology Company, Nanning, Guangxi, China) in the experimental field. Residues of fluopyram in eggplant fruit were analysed by gas chromatography (GC), using a Spherisorb DB-17 column (Agilent). Detection was performed with an ECD detector using the fluopyram standard (purity was 99.4%, Ehrenstorfer GmbH Co.). A 41.7% fluopyram suspension concentrate (SC) was used in the field experiment (Bayer Crop Science Co., Ltd.), and the other reagents were of analytical grade (Guangzhou Chemical Reagents Factory Co., China).

Site description

Field experiments were carried out at Jinling Village (22.92° N, 108.05° E) in Nanning, Guangxi, China, where a hot climate exists with an average temperature of 22.4℃. In May, the precipitation was 29.4 mm, and the total illumination was 109.6 h; in August, the average temperature was 28.0℃, the total precipitation was 124.9 mm and the total illumination was 171.4 h. The climatic characteristics of

the experimental area were suitable for propagation of root-knot nematodes. The total planting area was approximately 4.2 ha. The soil type was loamy with a soil pH of 7.8. Fertilizer applications and other agronomic practices were carried out regularly as needed.

Experimental design and treatment application

The field experiment was conducted in adjacent fields in May and August 2018. The whole field was divided into two equal portions for separate experiments during May and August. Each experimental plot was 300 m^2, which was divided into 3 random treatment plots, and the experimental plots were separated from each other by a 70 m^2 protective plot.

In the field, a drip tape of chemigation watered one adjacent row of eggplant in the boll stage and was covered with black, virtually impermeable film. Eggplants were separated from each other by 0.6 m. The chemigation system was based on the available drip irrigation system with an added bucket for applying fluopyram. The principle of operation was siphonage. After germination of the 4th true leaf, 41.7% fluopyram SC was applied only once in low, moderate and high doses (40, 60 and 80 $g \cdot ha^{-1}$, LD, MD and HD, respectively) with low and high irrigation water volumes (100 and 200 $L \cdot ha^{-1}$, LIWV and HIWV, respectively) at low and high flow velocities (1 and 2 $L \cdot h^{-1}$, LFV and HFV, respectively) per the appropriate procedures [17]. Fluopyram was applied with 75 and 150 $L \cdot ha^{-1}$ irrigation water for each water volume treatment, then was applied with 25 and 50 $L \cdot ha^{-1}$ irrigation water to rinse the tape. The drip tape was cut at the end and closed using end caps.

The sampling area was divided into 7 spots according to the distance from the spot to the dripper. Then, spot 1 (a circular area with a radius of 6 cm from the dripper, centre area, CA); spots 2 and 3 (torus-shaped areas with distances to dripper of 6 cm and widths of 4 cm, close-distance area, CDA); spots 4 and 5 (torus-shaped areas with distances to dripper of 10 cm and widths of 4 cm, mid-distance area, MDA); and spots 6 and 7 (torus-shaped areas with distances to the dripper of 14 cm and widths of 4 cm, long-distance area, LDA) were established.

Control efficacy for RKN

On each sampling date (7, 15 and 30 days after treatment, DAT), 500 g soil samples were collected consisting of soil samples from 7 sampling spots around a plant. The separation method for RKN used a Baermann funnel, described by [18]. Soil samples from each spot were separated and replicated 3 times. RKN populations were counted by microscope. RKNs were considered to be dead if they did not respond to being touched with a small probe. The RKN efficacy can be described by the following equations based on [19]:

$$\text{Corrected mortality (\%): } m = (m_t - m_c) / (1 - m_c) \times 100 \quad (1)$$

$$\text{General mortality (\%): } M = \sum_{i=1}^{4} m_i \cdot s_i / S \quad (2)$$

where m_t is nematode mortality (%) from fluopyram treatment, and m_c is the blank control mortality (%); m_i is nematode mortality (%) from different sampling areas; s_i is the area (cm^2) of different sampling areas, i = 1,2,3 and 4, representing the centre, close-distance, mid-distance and long-distance areas; and S is the area (cm^2) of the entire sampled area.

Functional diversity of soil microbial community determination

BIOLOG Eco plates (BIOLOG Inc., Hayward, USA) were used to study the substrate utilization patterns of soil microbial communities [20]. On each sampling date (3, 7, 14, 21 and 30 DAT), 500 g soil samples were randomly collected for each treatment. The AWCD, Shannon index, Simpson's diversity index and the McIntosh index were determined by calculating the mean of the absorbance value for every well after 96 h incubation, which corresponded to the time of maximal microbial growth in the BIOLOG Eco plates as determined by a BIO-TEKElx 808 automated micro plate reader (BIOLOG Inc., Hayward, USA) [15].

$$\text{AWCD} = \sum OD_i / 31 \quad (3)$$

$$\text{Shannon index: } H' = -\sum P_i \times \ln(P_i) \quad (4)$$

$$\text{Simpson index: } D = \sum [n_i (n_i - 1) / N (N-1)] \quad (5)$$

$$\text{McIntosh index: } U = \sqrt{\sum (n_i)^2} \quad (6)$$

where OD_i is the optical density value from each well after subtracting the value of the blank (water). pi is the ratio of microbial activity on each substrate (OD_i) to the sum of the microbial activities on all substrates, ΣOD_i. n_i is the absorbance value, N is the total absorbance value for all wells, and the Simpson index is expressed as the reciprocal (1/D).

Soil enzyme activities determination

The activities of three soil enzymes (urease, *β*-glucosidase and alkaline phosphatase) were determined to evaluate the ecotoxicology of fluopyram. On each sampling date (3, 7, 14, 21 and 30 DAT), 500 g soil samples were randomly collected for each treatment and were screened for soil enzyme analysis. The operation followed the instructions of a soil enzyme assay kit (SOLABIO, CO). Finally, the mixtures were measured with a spectrophotometer (UV-2600, Shimadzu, Japan) at 400 nm (soil *β*-glucosidase), 630 nm (soil urease) and 660 nm (soil alkaline phosphatase).

$$\text{Inhibition rate：} \quad p_i = (1 - a_i / a_c) \times 100\% \qquad (7)$$

where p_i is the inhibition rate of fluopyram on soil enzyme activity; a_i is the activity of a soil enzyme after treatment from different soil sampling spots, from spot 1 to 7; and a_c is the activity of a soil enzyme in the control.

Fluopyram residues in eggplant fruit

The determination method was based on [21] with some modifications The eggplant fruit samples were collected and crushed at 30 DAT, and a 10.0 g sample was weighed and placed in a centrifugal tube. A volume of 20.0 mL acetonitrile with 4.0 g NaCL was added and mixed for 1 minute. After 30 minutes, the samples were centrifuged at 1000 xg for 5 minutes. The supernatant was collected and dried by rotary evaporation at 70℃, then 2.0 mL n-hexane was added and covered. A Florisil SPE Column was leached with 3.0 mL leachate (n-hexane: acetone = 7:3, v/v), and then 3.0 mL n-hexane and the sample solution were added. A 10 µl sample was injected into the GC system for measurement.

Statistical analysis

All quantitative data were presented as the mean ± SE of at least three independent experiments by Tukey's test to determine the differences using SPSS 20.0. A *P*-value of 0.05 was considered to be statistically significant.

Results

Exploration of the effect factor for the corrected mortality of RKN

The corrected RKN mortalities resulting from different treatments are presented in Table 1a. The results from the field experiments showed that the corrected RKN mortalities for different sampling areas were related to the distances from drippers. The general RKN mortalities are presented in Table 1b. The corrected mortalities in the LD group were significantly lower than those in other dose groups for each sampling day. There was a significant difference between the corrected mortalities in the HIWV group and those in LIWV group at the same fluopyram dose and for the same flow velocity. The corrected mortalities in the HFV group were greater than those in the LWV group, but the difference was insignificant. In the HIWV and HFV treatment groups, the general mortalities for the 60 $g{\cdot}ha^{-1}$ fluopyram treatment were 56.55%, 62.60% and 69.51% at 7, 15 and 30 DAT, respectively.

Table 1 (a)

Corrected mortalities from fluopyram applied by chemigation to control RKN in the field.

DF	IWV	FV	Days after treatment											
			7				15				30			
			CA	CDA	MDA	LDA	CA	CDA	MDA	LDA	CA	CDA	MDA	LDA
40	100	1	43.42±2.22c	30.74±2.59c	20.66±3.47d	14.04±4.15g	51.57±2.46d	37.1±1.88h	28.24±1.99d	19.68±4.89d	62.56±2.72c	51.18±2.13d	36.85±1.62e	31.75±2.32d
		2	46.03±2.22c	31.72±2.38c	20.99±4.23d	14.49±4.73g	52.17±1.77d	38.88±2.04fg	29.43±2.14d	20.87±4.04d	63.03±1.82c	52.13±1.58d	38.15±2.63e	29.72±2.51cd
	200	1	43.42±2.21c	34.65±2.64c	24.24±2.45d	16.76±4.45g	52.76±3.47d	44.19±4.13efg	36.51±3.12c	23.52±3.21d	62.56±3.03c	58.29±2.09c	41.94±3.03de	34.83±2.43cd
		2	43.42±2.09c	35.30±2.35c	25.87±3.40d	19.36±2.78fg	52.17±0.51d	45.37±2.23efg	38.58±2.60c	27.07±4.57cd	63.03±2.74c	59.00±2.53c	44.05±3.38de	36.97±4.11cd
60	100	1	59.68±3.09b	44.73±3.39b	34.97±2.99c	24.79±1.81ef	65.75±2.26bc	50.69±1.83def	39.76±1.71c	32.97±2.27bc	72.92±0.91b	56.62±3.34cd	47.39±3.20d	38.63±4.08 bc
		2	60.33±2.68b	45.38±3.03b	35.95±3.54c	27.17±3.75de	66.34±4.02bc	52.46±4.57de	41.53±4.23c	32.97±3.51bc	73.41±3.24b	63.98±2.28bc	47.39±4.05d	39.81±3.03bc
	200	1	59.68±3.71b	48.95±3.15b	42.45±2.55b	37.07±3.30bc	65.16±3.10c	58.07±3.27bcd	49.51±2.40b	40.94±2.23ab	73.41±2.85b	67.06±2.95b	55.45±4.38c	44.79±2.51b
		2	60.98±1.84b	50.25±2.45b	44.08±1.84b	38.22±1.94bc	66.93±3.34bc	58.36±3.21bcd	49.51±1.41b	43.01±4.52a	73.91±2.97b	68.24±2.41b	56.40±2.21c	44.79±2.35b
80	100	1	70.74±2.22a	55.78±3.11a	43.02±1.35b	30.97±2.42cde	75.20±2.46ab	65.45±3.28bc	52.76±1.49b	40.65±2.23ab	82.46±1.62a	73.46±1.52a	63.74±3.74b	51.42±2.72a
		2	70.09±1.30a	58.06±2.13a	42.77±3.99b	32.69±4.37cd	76.97±3.48a	65.15±3.10abc	50.98±3.72b	40.94±0.57ab	82.46±2.10a	74.88±2.01a	66.11±1.28ab	52.84±2.35a
	200	1	71.39±1.50a	60.01±1.54a	51.55±2.30a	44.08±4.11ab	76.97±1.72a	68.70±3.77ab	60.43±1.97a	45.96±1.83a	81.99±1.97a	78.20±2.32a	69.91±2.55ab	54.50±2.02a
		2	70.09±1.68	60.66±2.14a	51.55±2.30a	46.68±1.20a	76.38±3.47a	70.18±3.40a	61.61±2.34a	47.74±3.96a	84.36±1.42a	78.88±3.52a	71.56±1.60a	56.40±3.74a
	df		47	95	95	95	47	95	95	95	47	95	95	95
	F		23.806	35.998	26.447	21.579	11.412	21.449	32.902	12.098	16.130	30.419	34.727	19.367
	P		< 0.001	< 0.001	< 0.001	< 0.001	< 0.001	< 0.001	< 0.001	< 0.001	< 0.001	< 0.001	< 0.001	< 0.001

DF represents the dose of fluopyram ($g \cdot ha^{-1}$), IWV represents irrigation water volume ($L \cdot ha^{-1}$), and FV represents the flow velocity ($L \cdot h^{-1}$). All data represent means ± SE. Values followed by different letters in the same column indicate significant differences ($P < 0.05$) according to Tukey's test.

Table 1 (b)

General mortalities of fluopyram applied by chemigation to control RKN in the field.

DF	IWV	FV	Days after treatment		
			7	15	30
40	100	1	37.75 ± 1.93d	45.46 ± 2.00d	56.64 ± 1.86d
		2	39.80 ± 1.64d	46.27 ± 1.76d	57.25 ± 1.27d
	200	1	38.70 ± 128d	48.00 ± 2.28d	58.12 ± 1.97d
		2	39.12 ± 1.58d	48.28 ± 3.80d	58.84 ± 2.24d
60	100	1	53.21 ± 2.31c	59.26 ± 1.74c	67.00 ± 1.04c
		2	53.97 ± 2.31c	59.99 ± 2.55c	67.51 ± 2.23c
	200	1	55.17 ± 2.83c	61.11 ± 2.34bc	68.95 ± 1.60c
		2	56.55 ± 1.33bc	62.60 ± 2.00abc	69.51 ± 0.57bc
80	100	1	63.60 ± 1.65ab	69.47 ± 1.75abc	77.42 ± 1.24ab
		2	63.46 ± 1.04ab	71.15 ± 0.80ab	77.90 ± 1.64a
	200	1	66.21 ± 1.03a	72.21 ± 1.29a	78.39 ± 1.60a
		2	65.60 ± 1.05a	72.18 ± 2.50a	80.48 ± 1.13a
	df		47	47	47
	F		40.611	22.812	31.004
	P		< 0.001	< 0.001	< 0.001

All data represent means ± SE. Values followed by different letters in the same column indicate significant differences ($P < 0.05$) according to Tukey's test.

The mortalities for the 80 g·ha^{-1} treatment were 65.60%, 72.18% and 80.48%, respectively, on the mentioned dates**.** Although fluopyram at the HD level exhibited better control of RKN, it also substantially affected soil community structures (Fig 1).

Fig 1. Effects of different rates of fluopyram on the functional diversity of the soil microbial community. The dashed line represents the average percentage of control. "*" represents significant differences between two-time treatments and control at each time as measured by Tukey's test (P < 0.05).

Analysis of the main effects and interactions showed that the times (df = 3; F =2.013; P < 0.0001) and irrigation water volumes (df = 2; F = 1.080; P < 0.0001) were significant factors contributing to the control efficacy for RKN except for the fluopyram rate, while a flow velocity (df = 2; F = 0.495; P < 0.0001) did not exhibit a substantial effect for controlling RKN. The higher water volume (200 L·ha^{-1}) and higher flow velocity (2 L·h^{-1}) were appropriate parameters for chemigation of fluopyram to control the root-knot nematode.

Control efficacy of fluopyram on eggplant root-knot nematodes

There were no significant differences between the RKN mortalities from different seasons (Fig 2). The corrected RKN mortality was maintained at 60.98% from 7 to 30 DAT with a peak of 73.91% at 30 DAT in the centre area in May, while RKN mortality was maintained at 61.20% from 7 to 30 DAT with a peak of 75.35% at 30 DAT in August. The RKN mortalities for different soil locations exhibited a normal distribution tendency, and the most efficient control was observed at the centre area under the dripper and gradually decreased with distance from centre area.

Fig 2. Corrected RKN mortality of fluopyram applied by chemigation under the most effective parameters on root-knot nematodes in a two-time field experiment. The blue figure represents the corrected RKN mortality in field experiments in May, 2018; the orange figure represents the corrected RKN mortality in the adjacent experimental field in August, 2018.

Effects of fluopyram on the functional diversity of the soil microbial community

There were similar effects between the data from the two experiments that could be combined for analysis. As shown in Fig 3, there were significant differences between treatment and control in the Shannon and McIntosh indexes at 30 DAT. The AWCDs of the treatments were close to the control level during the experimental period except for those of the first treatment at 7 and 30 DAT. The Shannon index of treatments decreased by an average of 5.85% at 30 DAT. The Simpson index for the treatments increased from 7 to 30 DAT, but the differences between the treatments and control were insignificant. The McIntosh index in the treatment groups was significantly higher than in the control group at 30 DAT, and the mean increase was 11.41%. As described above, fluopyram changed the soil microbial functional diversity.

Fig 3. Variations in the soil microbial community index as affected by fluopyram. The dashed line shows the average percentage of control. The blue square represents the relative value of treatment on root development in the experimental field in May 2018; The orange circle represents the relative value of treatment on root development in the adjacent experimental field in August, 2018. "*" represents significant differences between treatment and control according to Tukey's test ($P < 0.05$).

Effects of fluopyram on the activity of soil enzymes

Soil enzymes, especially *β*-glucosidase, have a critical role in C mineralization. Similarly, urease and alkaline phosphatase also play critical roles in the N and P cycles, respectively [22]. Therefore, soil enzyme activity could be an indicator of soil biological activity [17,23]. The responses of soil enzyme activities, including urease, *β*-glucosidase and alkaline phosphatase, after fluopyram application by chemigation are shown in Fig 4. The activity of soil urease in the treatments showed significant changes relative to the control at 21 DAT, particularly in the centre, close and mid-distance areas (from spot 1 to spot 5). The effect of fluopyram on soil urease showed an overall distinct decrease at 7 DAT,

while the soil urease activity of the treatments returned to the control level at 30 DAT. Fluopyram significantly inhibited the activity of soil β-glucosidase in the centre area at 3 DAT. The effect on the activity of soil β-glucosidase in the close-distance area increased slightly with the diffusion of fluopyram. The soil β-glucosidase activity for all areas recovered gradually from 14 to 30 DAT, and no significant differences were observed between control and treatments at 30 DAT. However, a slight increase in the centre and close-distance areas was observed from 3 to 7 DAT.

Fig 4. Effects of fluopyram on soil enzyme activity. (a) Soil urease. (b) Soil- β - glucosidase. (c) Soil alkaline phosphatase. Each column is the average value of the triplicates. The standard deviation is illustrated using an error bar. The significant differences among different sampling spots are illustrated using different letters above the columns at the $p < 0.05$ level via the least significant difference (LSD) test. Significant differences between treatment and control are illustrated using a "*" symbol above the columns at the $p < 0.05$ level via Tukey's test.

Fluopyram residues in eggplant fruit

Reliable linearity, y = 19612 x -1766.2, was achieved with fluopyram standard dosages in the range from 0.05 to 1.00 μg/ml with a correlation coefficient (R^2) = 0.9878 for fluopyram in all cases. The recovery rates ranged from 80.55% to 84.76%, and the relative standard deviations (RSD) ranged from 3.74% to 10.80%. In all cases, the results from the recovery tests were acceptable and confirmed that the method was sufficiently reliable for fluopyram analysis in this study. The terminal residue (30 DAT) of fluopyram was 0.076 mg·kg^{-1}, which was below the maximum residue limit of fluopyram in eggplant fruit, 0.9 mg·kg^{-1} [24].

Discussion

Appropriate application of nematicide can improve efficiency, reduce the dose of nematicides and consequently, reduce the costs. In this experiment, fluopyram was applied at a dose of 60 g • ha^{-1} with 200 L • ha^{-1} irrigation water and a 2 L • h^{-1} flow velocity and showed substantial control of root-knot

nematodes, resulting in 69.51% and 70.22% general mortality at 30 DAT for two continuous field experiments. The results were similar in beans when fluopyram was applied at a dose of 91.74 g • ha^{-1} below the seed in furrows, for which the control efficacy was 88.34% for RKN. Likewise, the control efficacy of 10 mg • L^{-1} abamectin SC was 74% on RKN [19]. The RKN mortality from fosthiazate for potato cyst nematodes was 74.85% [25]. Fluopyram was no less effective than other popular nematicides. Hence, the control efficacy of eggplant RKN for fluopyram applied via chemigation seemed acceptable when compared to other popular nematicides. The higher water volume treatment produced greater mortality than the lower volume treatment and spread fluopyram more uniformly and widely. The volume weight of tested soil was 1.05 g • cm^{-3}, and the soil permeability was 0.36 L • h^{-1}. When the flow velocity was faster than the soil permeability, chemigation reduced the downward loss of soil moisture and increased the horizontal motion of fluopyram in the irrigation water. This pattern was in accordance with [26,27]. These results suggested that fluopyram applied by chemigation systems is highly promising.

Many researchers have recently found that pesticides play a large role in ensuring and improving food production, but they also bring many negative impacts to the surrounding environment and to ecosystems [28,29]. As described above, fluopyram was initially developed as fungicide and was confirmed to change soil microbial communities [15]. Furthermore, changes in the soil environment caused by fungicides usually led to reductions in the abundance and diversity of microorganisms [16]. High rates of fluopyram had adverse effects on overall soil microbial activities and changed the soil microbial biota structure and its function due to its toxicity on natural systems [30], while the differences between lower-rate treatments and the control were found to be insignificant. The probable reason for this might the fact that the degradation started soon after the application of fluopyram at low dosages, and hence, the microbial community was restored and gradually reached a common level.

Soil urease enzyme activity is associated with microbial abundance [29]. In field experiments, the activity of soil urease was inhibited by fluopyram during the early and mid-periods but resumed at later periods. This dynamic process coincided with the AWCD of the microbial community. Soil *β*-glucosidase is one of the hydrolytic enzymes that hydrolyses cellulose to glucose. Soil phosphatase plays a key role in hydrolysing organic phosphate to inorganic form, thereby enhancing the supply of soil phosphorus [30]. In this study, the activity of soil alkaline phosphatase

seemed to be least sensitive to fluopyram. A correlation analysis showed that the fluopyram effects on soil enzyme activity were positively correlated to distance from the irrigation drippers. According to this discovery, eggplant should be planted in the close-distance area where acceptable control efficacy on the root-knot nematode is obtained and fewer negative effects on soil enzymes are induced.

Microorganisms are major soil decomposers that drive key processes, and fluopyram residues depended mostly on soil type and the application method used [31]. High-rate fluopyram restricted the degrading activity of soil microorganisms [32]. Fluopyram and its major metabolite, benzamide, are less likely to enter the food chain through intake from crops [33].

Conclusion

The study suggested that chemigation system is beneficial not only for farming water usage in karst landscape in Guangxi, but also in controlling soil-disseminated disease efficaciously, stably and safely.

Acknowledgements

This work was generously supported by the Key Research and Development Program of Guangxi (Guike AB16380118).

Author Contributions

Conceptualization: Wenwei Tang. Funding acquisition: Dongqiang Zeng. Methodology: Wenwei Tang and Jinzhao Li. Validation: Jinzhao Li, Cancan Wang and Haiou Lin. Writing-Original Draft Preparation: Jinzhao Li. Writing-Review & Editing: Wenwei Tang and Saqib Hussain Bangash.

References

1. Cai P, Li Y, Liu D, Liu X, Liang G, Yang H, et al. Effect of rootstock solanum torvum on growth performance yield and quality of eggplant in summer and autumn. southwest China Journal of Agriculture Science. 2015; 28 (3) : 4–7. DOI: 10.16213/j.cnki.scjas.2015.03.052
2. Guo JH, Li K, Jiang CH, Xu Q, Xie YS, Wang JS, et al. Evaluation of root-knot nematode disease control and plant growth promotion potential of biofertilizer Ning shield on trichosanthes kirilowii in the field. Braz J Microbiol. 2017; 49 (2): 232–239.
3. Mo J, Wang Y, Hu F, Guo T. Diagnosis and control techniques of major diseases of eggplant in Guangxi. J Chang Vegatables. 2011; 18: 67–70. DOI:10.3865/j.issn.1001-3547.2011.18.023
4. Haase S, Marhan S, Hallmann J, Ruess L, Poll J, Kandeler E. Low amounts of herbivory by root-knot nematodes affect microbial community dynamics and carbon allocation in the rhizosphere. FEMS Microbiol Ecol. 2007; 62 (3): 268–279. DOI: 10. 1111/ j. 1574-6941. 2007. 00383.x
5. Colagiero M, Rosso LC, Ciancio A. Diversity and biocontrol potential of bacterial consortia associated to root- knot nematodes. Biol Control. 2018; 120: 11–16. DOI: 10. 1016/ j. biocontrol. 2017.07.010
6. Veloukas T, Karaoglanidis GS. Biological activity of the succinate dehydrogenase inhibitor fluopyram against Botrytis cinerea and fungal baseline sensitivity. Pest Manag Sci. 2012; 68 (6): 858–864. DOI:10.1002/ps.3241
7. Weber RWS, Entrop A, Goertz A, Mehl A, Jork O, Niedersachsen L, et al. Status of sensitivity of Northern German Botrytis populations to the new SDHI fungicide fluopyram prior to its release as a commercial fungicide. J Plant Dis Prot. 2015; 122 (2): 81–90. DOI:10.1007/bf03356535
8. Vitale A, Panebianco A, Polizzi G. Baseline sensitivity and efficacy of fluopyram against Botrytis cinerea from table grape in Italy. Ann Appl Biol. 2016; 169 (1): 36–45. DOI:10.1111/aab.12277
9. Faske TR, Hurd K. Sensitivity of Meloidogyne incognita and rotylenchulus reniformis to Fluopyram. J Nematol. 2015; 47 (4): 316–321.
10. Li YB, Hou JY, Xie DT. The recent development of research on karst ecology in southwest China. Sci Geogr Sin. 2002; 22 (3): 365–370. DOI:10.13249/j.cnki.sgs.2002.03.019
11. Yang ST, Tian L. Research on the application of soil water delamination equilibrium model in karst area. C ARSOLOGICA SIN ICA. 2005; 24 (3): 186–91. DOI:10.3969/J.ISSN.1001-4810.2005.03.004
12. Guangxi Statistical Bureau. Guangxi Statistical Yearbook 2018. Available from: http://data.cnki.net/Search/ReportPreview?filename=N2018110018000241
13. Wang J, Niu W, Li Y, Lv W. Subsurface drip irrigation enhances soil nitrogen and phosphorus metabolism in tomato root zones and promotes tomato growth. Appl Soil Ecol. 2018;124: 240–251. DOI:10.1016/j.apsoil.2017.11.014

13. Him T, Rudnick DR, Burr CA, Stockton MC, Werle R. Approaches to evaluating grower irrigation and fertilizer nitrogen amount and timing. Agric Water Manag. 2019; 213: 693–706. DOI:10.1016/j.agwat.2018.11.010

14. Castillo GX, Ozores-hampton M, Navia PA. Effects of fluensulfone combined with soil fumigation on root-knot nematodes and fruit yield of drip-irrigated fresh-market tomatoes. Crop Prot. 2017; 98:166–171. DOI:10.1016/j.cropro.2017.03.029

15. Zhang Y, Xu J, Dong F, Liu X, Wu X, Zheng Y. Ecotoxicology and environmental safety response of microbial community to a new fungicide fluopyram in the silty-loam agricultural soil. Ecotoxicol Environ Saf. 2014; 108: 273–280. DOI:10.1016/j.ecoenv.2014.07.018

16. Baćmaga M, Wyszkowska J, Kucharski J. The influence of chlorothalonil on the activity of soil microorganisms and enzymes. Ecotoxicology. 2018; 27 (9): 1188–1202. DOI:10.1007/s10646-018-1968-7

17. He J, Zhou L, Yao Q, Liu B, Xu H, Huang J. Greenhouse and field-based studies on the distribution of dimethoate in cotton and its effect on Tetranychus urticae by drip irrigation. Pest Manag Sci. 2018; 74: 225-233. DOI:10.1002/ps.4704

18. Ito T, Araki M, Komatsuzaki M. Field crops research no-tillage cultivation reduces rice cyst nematode (Heterodera elachista) in continuous upland rice (Oryza sativa) culture and after conversion to soybean (Glycine max) in Kanto, Japan. F Crop Res. 2015; 179: 44–51. DOI:10.1016/j.fcr.2015.04.008

19. Fang W, Ren Z, Wang Q, Ouyang C, Guo M, Li J, et al. Effect of soil fumigants on degradation of abamectin and their combination synergistic effect to root-knot nematode. PLoS One. 2018;13 (6): e0188245. DOI:10.1371/journal.pone.0188245

20. Girvan MS, Bullimore J, Pretty JN, Osborn AM, Ball AS. Soil type is the primary determinant of the composition of the total and active bacterial communities in arable soils. Appl Environ Microbiol. 2003; 69 (3): 1800–1809. DOI:10.1128/AEM.69.3.1800-1809.2003

21. Yu F, Fu P, Wang S, Wang X. Determination of fluopyram residues in tomato by gas chromatography. Agrochemicals. 2016; 4: 278–279.

22. Sahoo S, Adak T, Bagchi TB, Kumar U. Effect of pretilachlor on soil enzyme activities in tropical rice soil. 2017; 98 (3): 439–445. DOI:10.1007/s0012

23. Du Z, Zhu Y, Zhu L, Zhang J, Li B, Wang J, et al. Ecotoxicology and environmental safety eff ects of the herbicide mesotrione on soil enzyme activity and microbial communities. Ecotoxicol Environ Saf. 2018;164: 571–578. DOI: 10.1016/j.ecoenv.2018.08.075

24. 中华人民共和国国家卫生和计划生育委员会, 中华人民共和国农业部, 国家食品药品监督管理总局. 食品安全国家标准-食品中农药最大残留限量. 2016 p. 70.

25. Tobin JD, Haydock PPJ, Hare MC, Woods SR, Crump DH. Effect of the fungus Pochonia chlamydosporia and fosthiazate on the multiplication rate of potato cyst nematodes (Globodera pallida and G. rostochiensis) in potato crops grown under UK field conditions. Biol Control. 2008; 46 (2): 194–201. DOI:10.1016/j.biocontrol.2008.03.014

26. Li B, Ren Y, Zhang DX, Xu S, Mu W, Liu F. Modifying the formulation of abamectin to promote its efficacy on southern root-knot nematode (Meloidogyne incognita) under blending-of-soil and root-irrigation conditions. J Agric Food Chem. 2018; 66 (4): 799–805. DOI:10.1020/acs.jafc.7b04146

27. Lu H, Xu S, Zhang W, Xu C, Li B, Zhang D, et al. Activity of trans -2-Hexenal against southern root-knot nematode (Meloidogyne incognita) on tomato plants. J Agric Food Chem. 2017; 65 (3): 544-550. DOI:10.1021/acs.jafc.6b04091

28. Pereira JL, Antunes SC, Castro BB, Marques CR, Gonçalves AMM, Gonçalves F, et al. Toxicity evaluation of three pesticides on non-target aquatic and soil organisms: Commercial formulation versus active ingredient. Ecotoxicology. 2009;18 (4): 455–463. DOI:10.1007/s10646-009-0300-y

29. Sun X, Zhu L, Wang J, Wang J, Su B, Liu T, et al. Toxic effects of ionic liquid 1-octyl-3-methylimidazolium tetrafluoroborate on soil enzyme activity and soil microbial community diversity. Ecotoxicol Environ Saf. 2017; 135: 201–2088. DOI:10.1016/j.ecoenv.2016.09.026

30. Nannipieri P, Giagnoni L, Landi L, Renella G. Role of phosphatase enzymes in soil. In: Bünemann E, Oberson A, Frossard E, editors. Phosphorus in Action; 2011. pp. 215–243. DOI: 10.1007/978-3-642-15271-9_9

31. Eisenhauer N, Partsch S, Scheu S, Scherber C, Sabais ACW, Klier M, et al. No interactive effects of pesticides and plant diversity on soil microbial biomass and respiration. Appl Soil Ecol. 2009; 42 (1): 31–36. DOI:10.1016/j.apsoil.2009.01.005

32. Muñoz-leoz B, Ruiz-romera E, Antigüedad I, Garbisu C. Tebuconazole application decreases soil microbial biomass and activity. Soil Biol Biochem. 2011;43 (10): 2176–2183. DOI: 10.1016/j.soilbio.2011.07.001

33. Matadha NY, Mohapatra S. Uptake and distribution of fluopyram and tebuconazole residues in tomato and bell pepper plant tissues. Environ Sci Pollut Res. 2019; 26 (6): 6077-6086. DOI: 10.1007/s11356-018-04071-4

Figure 1

Click here to download Figure figure 1.png

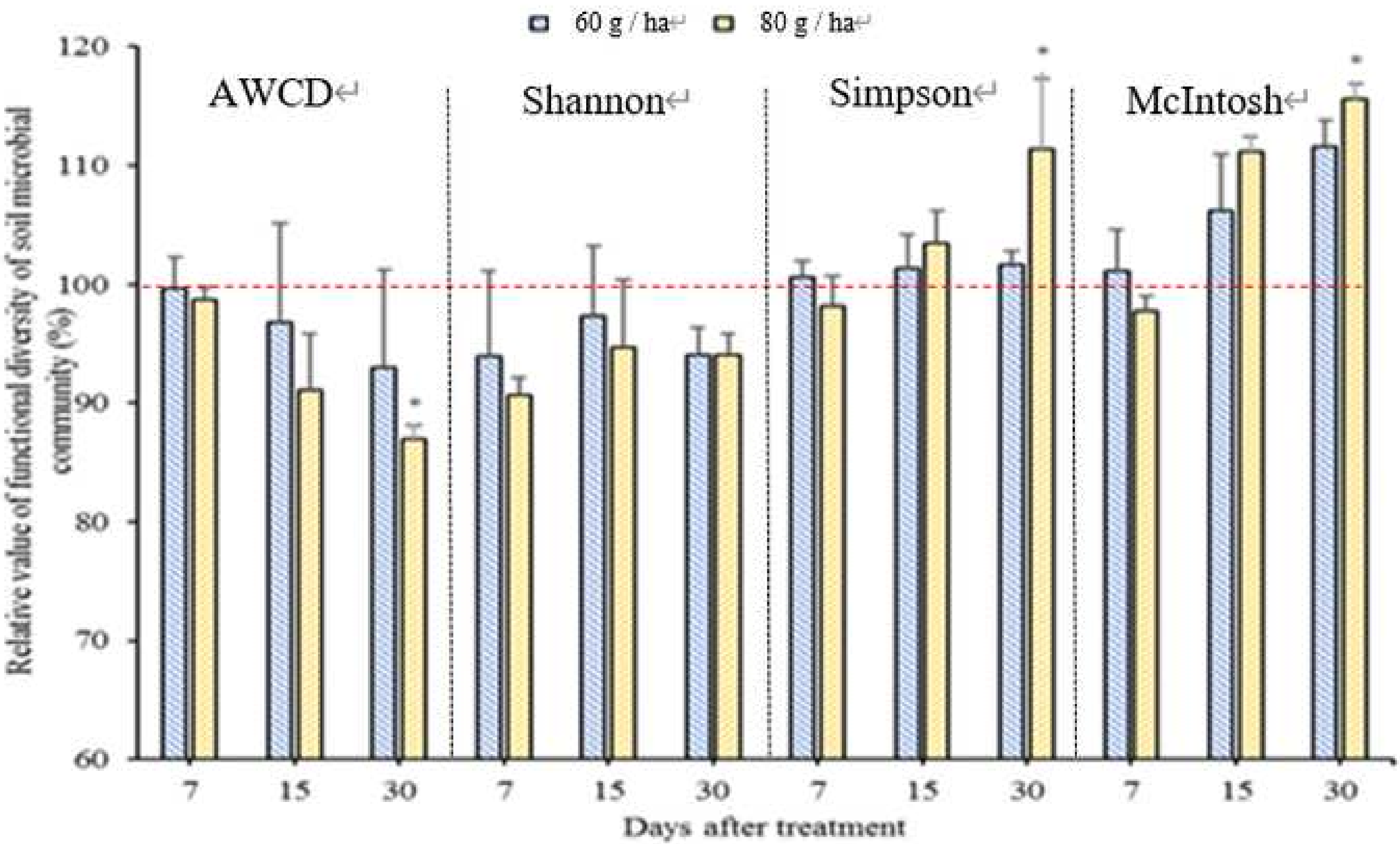

Figure 2

Click here to download Figure figure 2.jpg

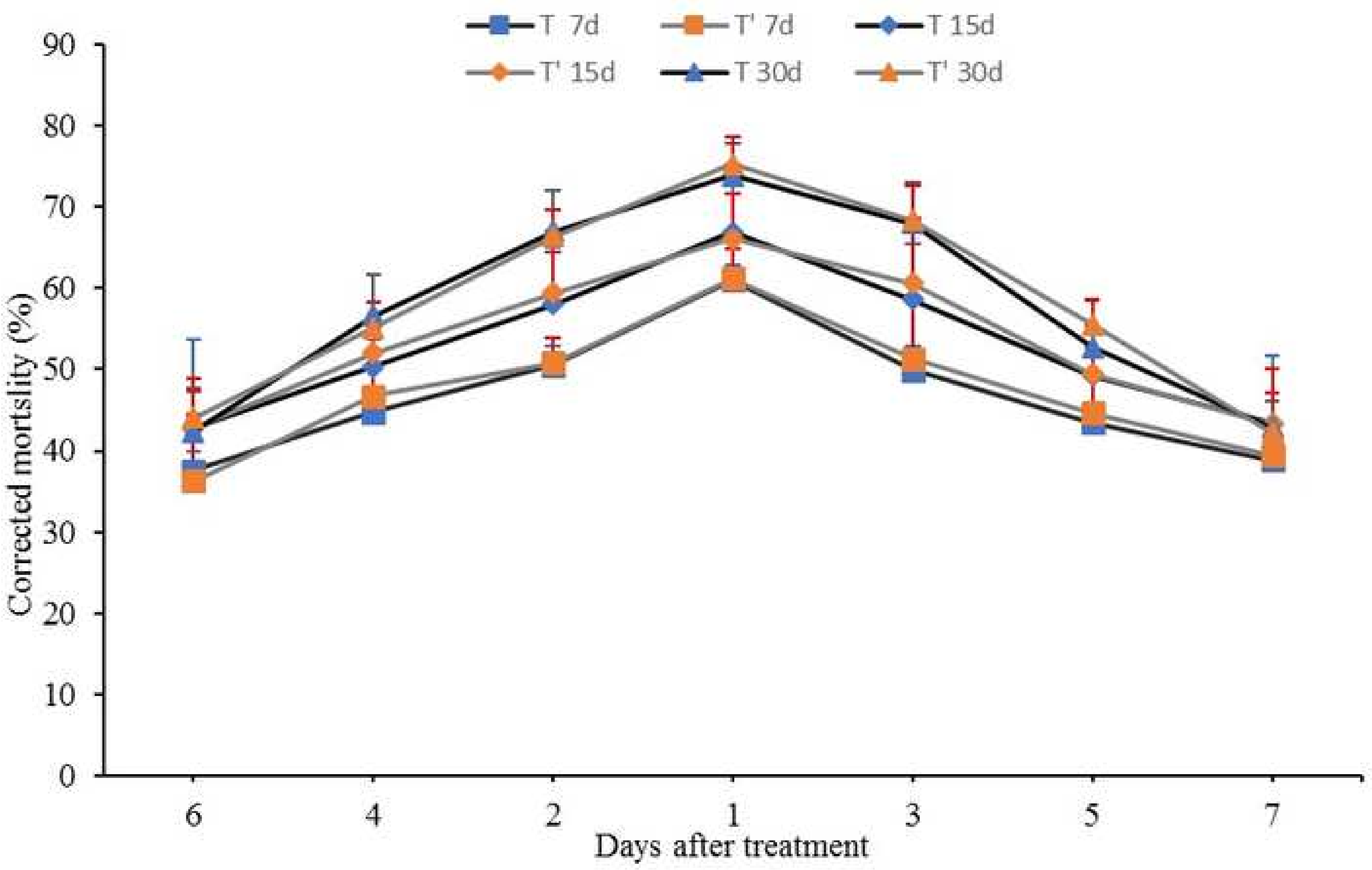

Figure 3 Click here to download Figure figure 3.jpg

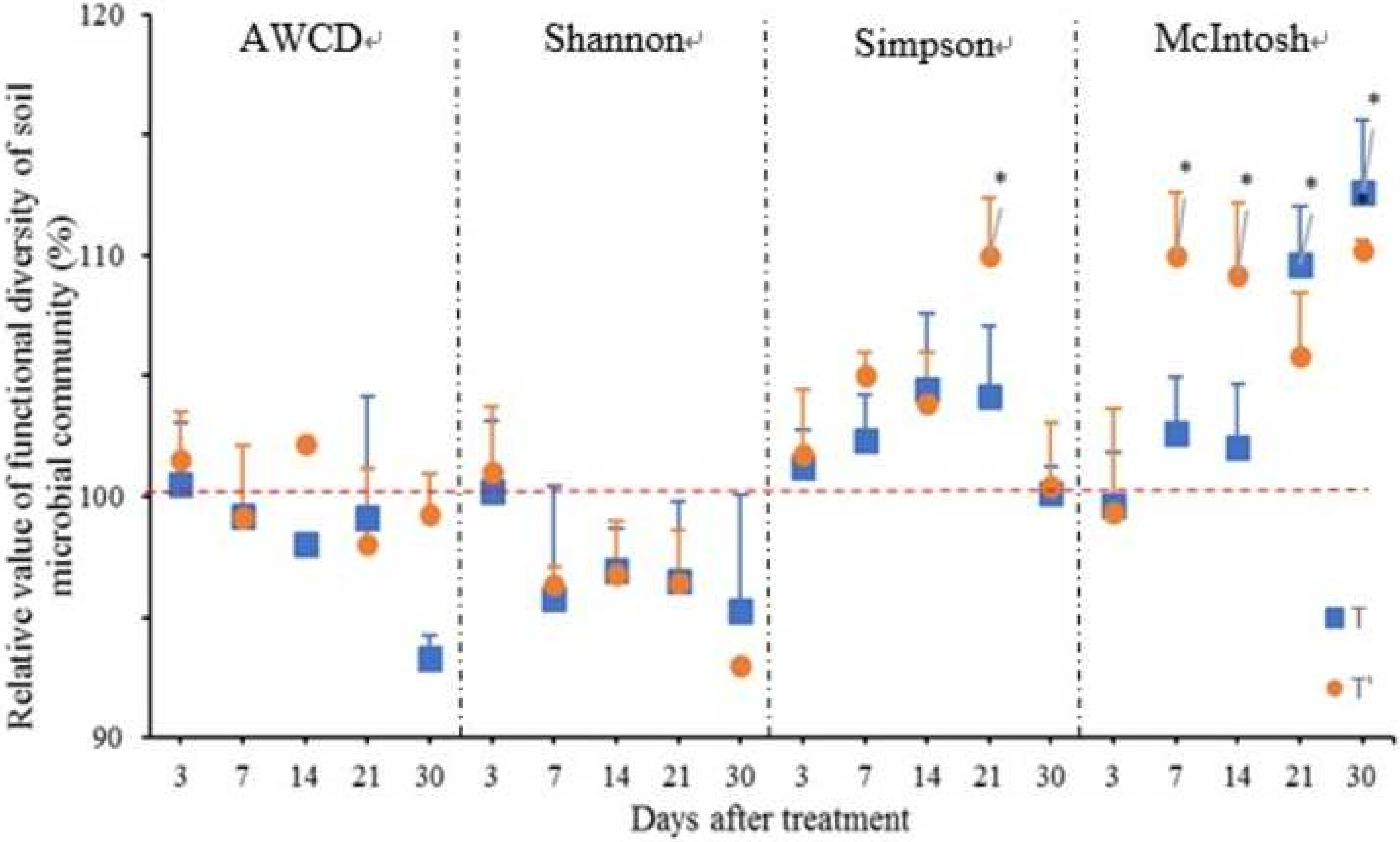

Figure 4

Click here to download Figure figure 4.jpg

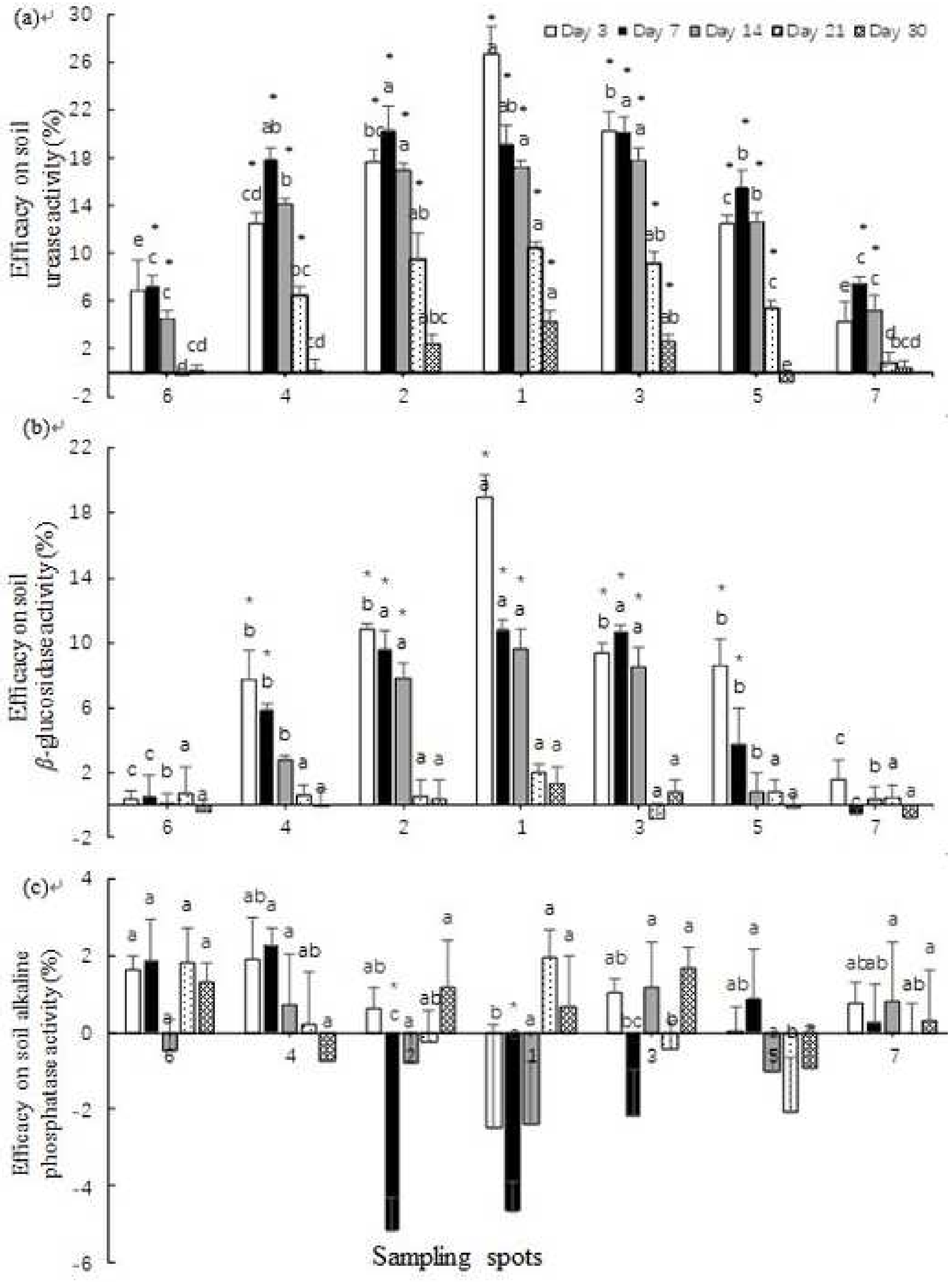

Supplement: Attachment — Submitted filename: PONE-D-20-00394_reviewer-1 (1).pdf [file pone.0235423.s001.pdf]
