# Supplementary material for: Efficacy of fluopyram applied by chemigation on controlling eggplant root-knot nematodes (Meloidogyne spp.) and its effects on soil properties
Source: PLoS One. 2020 Jul 6;15(7):e0235423. doi: 10.1371/journal.pone.0235423 (PMC7337292; doi:10.1371/journal.pone.0235423)
Supplement: Attachment — Submitted filename: Response to reviewers 1.docx [file pone.0235423.s002.docx]

Dear editor and reviewers:

Thank you very much for your kind review and advice concerning our manuscript. The comments are valuable and helpful for revising and improving our paper and have provided good guidance for our studies. We have substantially revised our manuscript (PONE-D-20-00394) after reading the comments provided by the four reviewers. We employed an English-language editing service, Wiley Editing Service, to publish our wording. We also expanded part of the experiment, providing details in the current version. In discussion, we compared present research with previous findings.

**Response to** Reviewer #1:

**Question 1:** Please follow instructions to author. Introduction is sufficient. Results and discussion are sufficient. but the titles of figures are not found above each figure. please correct the mistakes and comments present in the text.

**Answer:** Thanks very much for the Reviewer’s suggestion. We have corrected it according to the comments and matched figures and titles.

**Response to** Reviewer #2:

**Question 1:** The manuscript title in text is different. “Efficacy of fluopyram applied by chemigation on controlling eggplant root-knot nematodes (Meloidogyne spp.) and its effects on soil properties” or “Efficacy of fluopyram applied by chemigation on controlling eggplant root-knot-nematodes (Meloidogyne spp.)”

**Answer:** Thanks very much for the Reviewer’s suggestion. The title of manuscript is “Efficacy of fluopyram applied by chemigation on controlling eggplant root-knot nematodes (*Meloidogyne* spp.) and its effects on soil properties”.
**Question 2:** The manuscript needs to be edited for grammar and syntax.

**Answer:** The manuscript has been revised by an English speaker to publish our wording. If there are still some mistakes in the manuscript, please gently give the comments.

**Question 3:** The introduction is generally relevant but unsufficient information about the previous study findings for readers. Previous studies on fluopyram to control RKN should be given in introduction.

**Answer:** Thanks for the Reviewer’s suggestion. We have given previous studies on fluopyram to control RKN in introduction (Line 43-44).
**Question 4:** In discussion, presented data should be compared with previous findings. Some information is general concept. For example, last paragraph in discussion section.

**Answer:** Thanks for the Reviewer’s suggestion. We have compared presented data with previous findings in discussion section (Line 278-286, 296-301, 308-312).

**Response to** Reviewer #3:

**Question** **1:** The paper is difficult to read and the authors need to have this manuscript checked for English.

**Answer:** Thanks for the Reviewer’s suggestion. The manuscript has been revised by an English speaker to publish our wording. If there are still some mistakes in the manuscript, please gently give the comments.

**Question** **2:** There has been several recent publications on this nematicide, including on chemigation, and the authors should at least include some of this work in their references.

**Answer:** Thanks for the Reviewer’s suggestion. We have given previous studies on fluopyram to control RKN in introduction (Line 43-44) and compared presented data with previous findings in discussion section (Line 278-286).

**Question 3:** The experimental design is not clearly explained - not sure how many replications are there and the sampling procedure is confusing - would be good to include a figure / diagram to clarify the sampling methodology. Also, the Baermann funnel method used to extract nematodes (need to give the correct (=original) reference for this method) is typically used to collect active nematodes from soil, not sure why the authors used a probe (which probe?) to verify if nematodes were dead or alive (if nematodes were dead, it's probably because they died from lack of oxygen in the funnel neck because they were not tapped off soon enough). Overall, the methodology lacks detail and is difficult to read and understand.

**Answer:** We have made a picture to illustrate replications are there and the sampling procedure (Fig. 1). The probe we used is a medical probe in case of mimic death of nematodes. We added some details and adjusted word order to made method section convenient for reading and understanding.

**Question 4:** The data tables and figures are also very busy and again don't help the readability of the paper. It also seems like this is just a single field experiment - duplicating the trial would strengthen the data and make this more relevant.

**Answer:** We have matched tables and figures with titles for easy understanding. The whole field was divided into two equal portions for separate experiments during May and August. In first time experiment, we explored the effect factor for the corrected mortality of root-knot nematodes. To verify above findings and test the effect on soil properties, we conducted the second the experiment. We have corrected the improper expression in the manuscript.

**Response to** Reviewer #4:

**Question 1:** Actually I expected from the author to test remained nematode density in the soil on root knot disease parameters like disease incidence and disease severity compared with control, and also its effect on plant growth & yield parameters like fresh and dry weight of each of root and vegetative systems , plant hight, fruit weight and size ..etc, but unfortunately I didn't find such data. Also, it was better if the author takes more than one duration to determine Fluopyram residues in order to know the trend of product degradation exactly instead of consider one duration (30 DAT) for the purpose.

**Answer:** Thanks for the Reviewer’s suggestion. In this research, we determined 30 DAT duration to prove the safety of applying fluopyram by chemigation on eggplant root-knot nematode. The environmental security and formulations are also important before practical applications, and further research will be focused on these aspects.

We tried our best to improve the manuscript and made some changes in the manuscript. These changes will not influence the content and framework of the paper. And here we did not list the changes but marked in red in revised paper. We appreciate for Editors/Reviewers’ warm work earnestly and hope that the correction will meet with approval. Once again, thank you very much for your comments and suggestions.

Yours sincerely,

Wen-Wei Tang, Ph.D.

E-mail: [wenweitg@163.com](mailto:wenweitg@163.com)
